# Antifungal Activity against *Fusarium culmorum* of Stevioside, *Silybum marianum* Seed Extracts, and Their Conjugate Complexes

**DOI:** 10.3390/antibiotics9080440

**Published:** 2020-07-24

**Authors:** Laura Buzón-Durán, Jesús Martín-Gil, María del Carmen Ramos-Sánchez, Eduardo Pérez-Lebeña, José Luis Marcos-Robles, Ángel Fombellida-Villafruela, Pablo Martín-Ramos

**Affiliations:** 1 ETSIIAA, Universidad de Valladolid, Avenida de Madrid 44, 34004 Palencia, Spain; laura.buzon@uva.es (L.B.-D.); mgil@iaf.uva.es (J.M.-G.); eplebena@gmail.com (E.P.-L.); jlmarcos@iaf.uva.es (J.L.M.-R.); afv@pvs.uva.es (Á.F.-V.); 2 Servicio de Microbiología y Parasitología, Hospital Universitario Rio Hortega, SACYL, Calle Dulzaina, 2, 47012 Valladolid, Spain; mramoss@saludcastillayleon.es; 3 Instituto Universitario de Investigación en Ciencias Ambientales de Aragón (IUCA), EPS, Universidad de Zaragoza, Carretera de Cuarte, s/n, 22071 Huesca, Spain

**Keywords:** antifungal, candyleaf, deoxynivalenol, milk thistle, seedling blight, trichothecene

## Abstract

Fusarium head blight (FHB) is a disease that poses a major challenge in cereal production that has important food and feed safety implications due to trichothecene contamination. In this study, the effect of stevioside—a glycoside found in the leaves of candyleaf (*Stevia rebaudiana* Bertoni)—was evaluated in vitro against *Fusarium culmorum* (W.G. Smith) Sacc., alone and in combination (in a 1:1 molar ratio) with polyphenols obtained from milk thistle seeds (*Silybum marianum* (L.) Gaertn). Different concentrations, ranging from 32 to 512 µg·mL^−1^, were assayed, finding EC_50_ and EC_90_ inhibitory concentrations of 156 and 221 µg·mL^−1^, respectively, for the treatment based only on stevioside, and EC_50_ and EC_90_ values of 123 and 160 µg·mL^−1^, respectively, for the treatment based on the stevioside–polyphenol conjugate complexes. Colony formation inhibition results were consistent, reaching full inhibition at 256 µg·mL^−1^. Given that synergistic behavior was observed for this latter formulation (SF = 1.43, according to Wadley’s method), it was further assessed for grain protection at storage, mostly directed against mycotoxin contamination caused by the aforementioned phytopathogen, confirming that it could inhibit fungal growth and avoid trichothecene contamination. Moreover, seed tests showed that the treatment did not affect the percentage of germination, and it resulted in a lower incidence of root rot caused by the pathogen in Kamut and winter wheat seedlings. Hence, the application of these stevioside–*S. marianum* seed extract conjugate complexes may be put forward as a promising and environmentally friendly treatment for the protection of cereal crops and stored grain against FHB.

## 1. Introduction

Pathogenic microorganisms present in the environment are a menace for crops. At a worldwide level, diseases caused by plant pathogenic fungi significantly contribute to overall losses in terms of crop yield. To face this challenge, the use of traditional pesticides entails disadvantages related to handling hazards, cost, residues, and threats to human health and the environment. Consequently, European Directive 2009/128/EC established a framework to achieve their sustainable use, promoting integrated pest management and other alternative approaches such as the use of natural products [1].

Recent regulatory changes (PFC 6 within Regulation (EU) 2019/1009 of the European Parliament and of the Council of 5 June 2019) have led to a renewed interest in the valorization of the aforementioned natural products as antimicrobial agents, given that new preparations based on natural products are now contemplated as fertilizers (and not as phytosanitary products). In addition to posing a more environmentally friendly alternative to synthetic pesticides, they would also be suitable for application in organic Farming.

A promising source of bioactive products is *Stevia rebaudiana* (Bert.) Bertoni (commonly known as candyleaf), a perennial herbaceous plant of the Asteraceae family. Its leaves contain steviol glycosides (including stevioside, rebaudioside (A to F), steviolbioside, and isosteviol, among which stevioside, rebaudioside A, and rebaudioside C are the major metabolites [2]), polyphenols, chlorophylls, and carotenoids [3]. Such extractives have been suggested to exert beneficial effects on human health [4,5,6], and several studies have covered their antimicrobial activity [7,8,9,10]. Nonetheless, a literature survey revealed that their antifungal/fungistatic activity has been little studied [11,12,13,14]. With regard to phytopathogenic fungi, promising results were reported by Shukla et al. [15] and Ramírez et al. [16] against *Fusarium oxysporum*.

Specific and strong inhibitory activities against other *Fusarium* species have also been reported for phenolic and polyphenolic natural compounds [17], but their applicability is limited by their low solubility and bioavailability [18]. Nonetheless, they may be improved through the formation of inclusion compounds or conjugate complexes with, for instance, terpene glycosides [19], as shown by the enhanced in-vitro antifungal activities against *F. culmorum* attained with composites consisting of polyphenol inclusion compounds and silver nanoparticles [20]. The latter soil-borne fungus is one of the most important Fusarium head blight (FHB) causal agents (together with *F. graminearum* Schwabe [21]), and it poses problems in agriculture and the food industry as it not only leads to yield losses of up to 50% but also to trichothecene contamination [22,23,24]. 

The aim of the study presented herein is to investigate the antifungal activity of the aforementioned natural bioagents against *F. culmorum*, exploring the presumable synergist effects that could result from the combination of stevioside and milk thistle (*Silybum marianum* (L.) Gaertn) seed extracts, which have been reported to exhibit high total phenolic content [25].

## 2. Results

### 2.1. Sensitivity Tests

Two antifungal susceptibility testing experiments were conducted. The antifungal effect was first evaluated by the so-called “poisoned food method”, in which the inoculation is done by a mycelial disc (hyphae + spores) that is deposited in the center of the agar plate [26], and then confirmed by determining the inhibition rate percentage of the number of colonies formed, using an inoculum mainly composed of spores [27].

**Figure 1:**
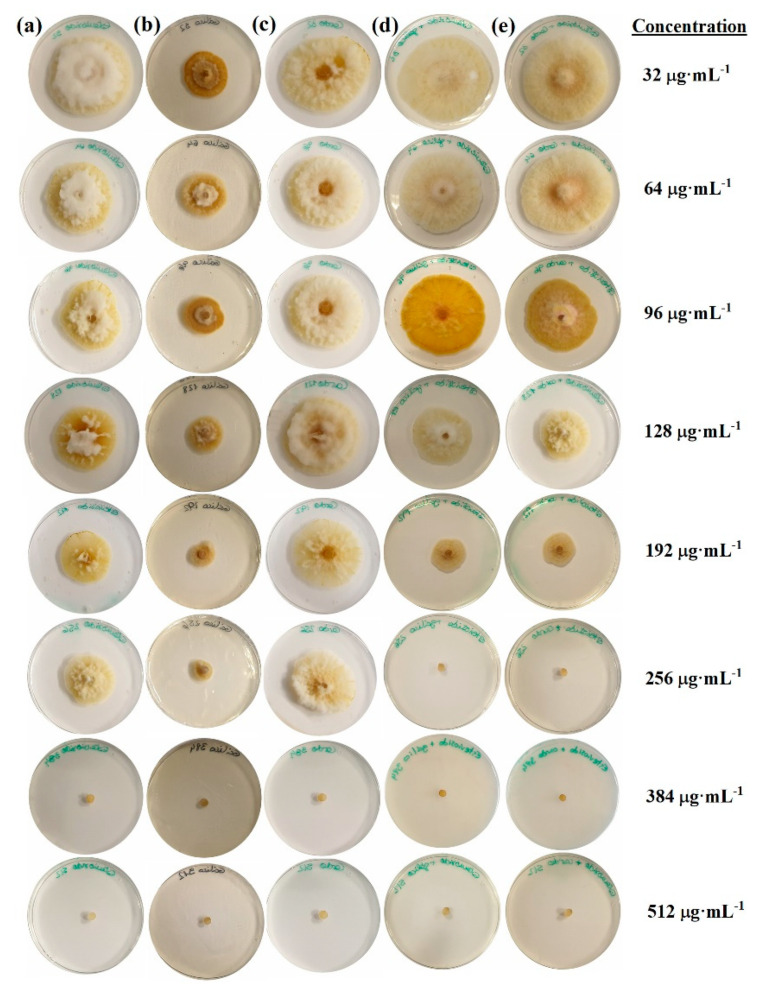
*F. culmorum* radial growth inhibition assays (“poisoned food method”) for the treatments based on (**a**) stevioside, (**b**) gallic acid, (**c**) milk thistle seed extracts, (**d**) stevioside–gallic acid, and (**e**) stevioside–milk thistle seed extracts. Only one replicate per treatment and concentration is shown. The control (PDA only) is not shown (radial growth = 60 mm).

**Figure 2:**
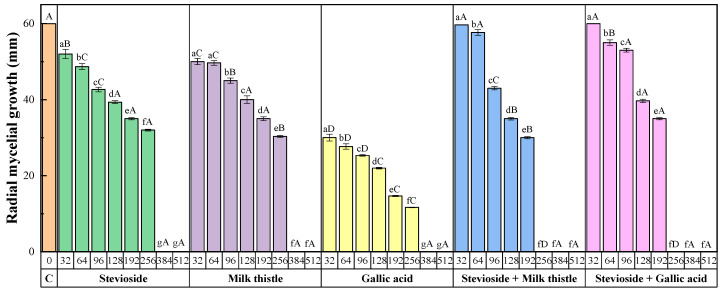
Radial growth values of *F. culmorum* in the presence of stevioside, gallic acid, milk thistle seed extracts, and the stevioside–polyphenol (i.e., stevioside–gallic acid and stevioside–milk thistle seed extract) conjugate complexes at different concentrations (expressed in µg·mL^−1^). Concentrations labeled with the same lowercase letters are not significantly different at *p* < 0.05 by Tukey’s test. Treatments labeled with the same uppercase letters are not significantly different at *p* < 0.05 at the indicated dose. All values are presented as the average of three replicates and two repeats. Error bars represent the standard deviation.

For the first in-vitro experiment, the mycelial growth inhibition obtained for each of the treatments and concentrations after 7 days is shown in Figure 1, and the values across the three replicates and two repeats are summarized in Figure 2. 

The increase in the treatment dose from 32 to 512 µg·mL^−1^ resulted in a reduction in the radial growth of the mycelium in all cases, with statistically significant differences amongst the various concentrations (Figure 2). Full inhibition was attained at the two highest doses (384 and 512 µg·mL^−1^) for all treatments. 

Statistically significant differences between treatments were observed at lower concentrations. However, those found at the 256 µg·mL^−1^ dose were particularly interesting: while the treatments based on stevioside–polyphenol conjugate complexes resulted in 100% inhibition, the treatments based on stevioside, milk thistle seeds extracts, and gallic acid led to inhibition percentages of 46.7%, 49.4%, and 80.5%, respectively. 

The efficacy of the treatments may also be compared by expressing the sensitivity test results in terms of the concentrations that reduced mycelial growth by 50% and 90% (EC_50_ and EC_90_, respectively), summarized in Table 1. The sensitivity of the isolate was very similar for the two treatments based on conjugate complexes, which showed a noticeably better inhibition than the stevioside or the polyphenols-only based treatments. According to the method of Wadley [28] for the quantification of the level of interaction, synergy factors (SFs) of 1.43 and 1.41 (>1.0) were obtained for the stevioside–milk thistle and the stevioside–gallic acid conjugate complexes, respectively, pointing to a synergistic interaction between the fungicides. 

**Figure 3:**
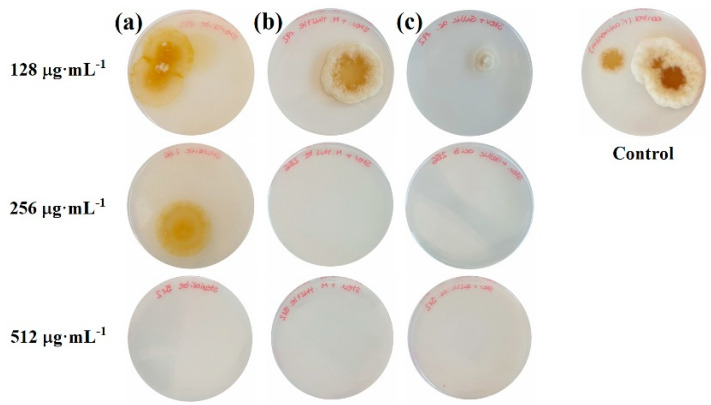
*F. culmorum* colonies formed after 5 days for the (**a**) stevioside, (**b**) stevioside–milk thistle seed extract, and (**c**) stevioside–gallic acid treatments. Only one replicate per treatment and concentration is shown.

In the second in-vitro antifungal susceptibility testing experiment, the number of colonies after incubation on the bioactive product-supplemented media was found to be clearly dependent on the bioactive product dose (Figure 3). Full inhibition was reached at 512, 256, and 256 µg·mL^−1^ for the stevioside, stevioside–milk thistle seed extract, and stevioside–gallic acid treatments, although the number of colonies was decreased with all concentrations in comparison with the control. Hence, the results on the inhibition of the number of colonies formed were consistent with the radial growth inhibition tests reported above.

### 2.2. Effect of the Stevioside−S. marianum Seed Extract Conjugate Complex on Deoxynivalenol Production

After incubation of the Kamut and winter wheat grain samples inoculated with *F. culmorum* for 28 days, complete inhibition was observed for the grains treated with the stevioside−milk thistle seed extract conjugate complex at a concentration of 512 µg·mL^−1^. No deoxynivalenol (DON) was detected (<LOD), while DON contents of 38.5 ± 2.1 and 53.2 ± 0.7 µg·g^−1^ were registered for the winter wheat and Kamut positive controls.

### 2.3. Seedling Tests

**Figure 4:**
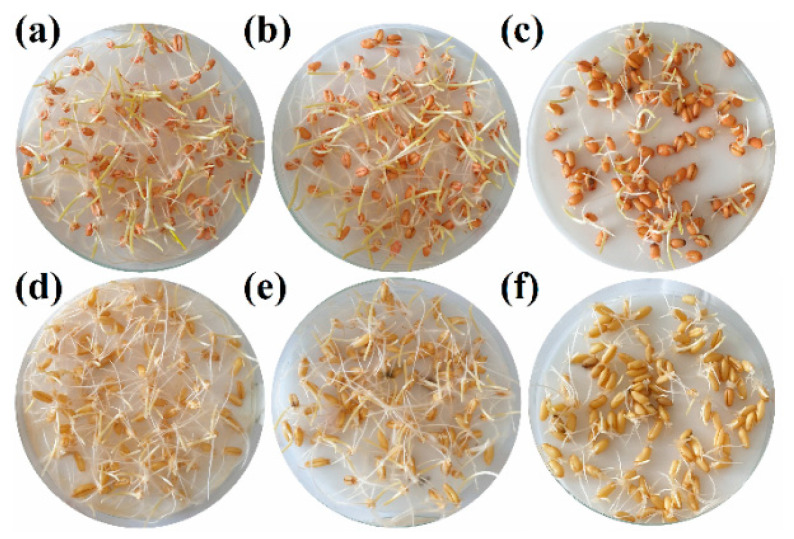
Germination tests for winter wheat seeds—(**a**) negative control; (**b**) treated seeds; (**c**) positive control—and Kamut seeds—(**d**) negative control; (**e**) treated seeds; (**f**) positive control—. Only one replicate is shown.

No significant differences were observed between the negative control and the noninoculated seeds treated with the conjugate complex (97% and 96% germination rates, respectively, for winter wheat seeds; 98% and 96% germination rates, respectively, for Kamut seeds) in the germination tests (Figure 4). The germination rate for the positive control (inoculated and not treated seeds) was noticeable lower (75% and 72% for winter wheat and Kamut, respectively), but it was clearly improved for the inoculated and treated seeds (92% and 85% for winter wheat and Kamut, respectively).

**Figure 5:**
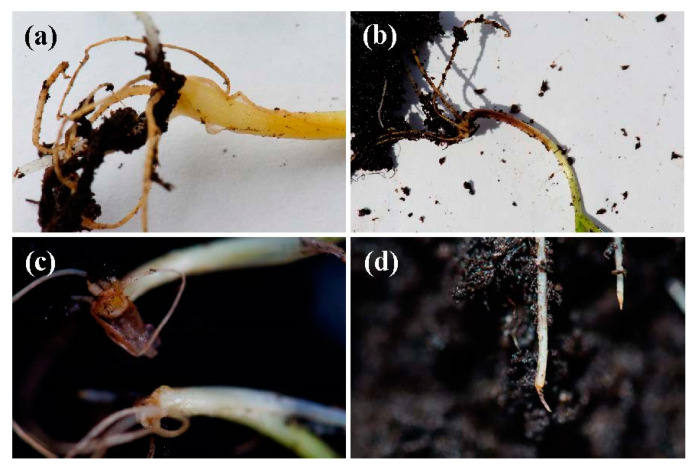
Different root rot symptoms observed in the artificially inoculated seedlings: (**a**) healthy coleoptile and roots; (**b**) necrotic lesions on the coleoptile; (**c**) necrotic lesions on the lower coleoptile, with scarce root development (top) vs. healthy seedling (bottom); (**d**) root apex browning.

With regard to root rot symptoms in the seedlings after 2 weeks, no symptoms were observed in the negative control for winter wheat and Kamut. In the positive control, some seedlings showed clear wilting and browning of coleoptiles and roots (Figure 5), particularly for Kamut, and the average disease severity was 10% for winter wheat and 14% for Kamut (with some seedlings reaching a 3 in the 0 to 5 degree of attack scale). In the artificially inoculated seedlings treated with the conjugate complex, the average disease severity was 4% in both cases. In other words, the disease index (DI) was reduced from 50% to 20% for winter wheat and from 35% to 20% for Kamut. Consequently, the control efficacy of the treatment could be deemed as moderate–high (60% and 71.4% for winter wheat and Kamut, respectively). 

Regarding the activities of some enzymes used as stress markers, namely, guaiacol peroxidase (POD) and ascorbate peroxidase (APX), or involved in defense-induced metabolic changes, namely, polyphenol oxidase (PPO) and phenylalanine ammonia lyase (PAL), no statistically significant differences were observed between treated and nontreated artificially inoculated seedlings for winter wheat or Kamut.

## 3. Discussion

### 3.1. Efficacy of the Treatments

The stevioside-only treatments showed high inhibitory activity, comparable to that of the polyphenol-only based treatments. This finding supports the antifungal activity of stevia leaves advocated by Shukla et al. [15], which would mainly be ascribed to stevioside and, to a lesser extent, rebaudoside and docosahexaenoic acid [29]. These authors found a MIC of 2 mg·mL^−1^ for *F. oxysporum*, much higher than the one reported herein. Ramírez et al. [16] evaluated the antifungal activity of extracts of different polarity obtained from *S. rebaudiana* leaves against *F. oxysporum* as well, finding that the hexane extract at a concentration of 833 ppm (five times higher than the one reported herein for stevioside) only inhibited mycelial growth by around 50%. The differences may be ascribed to both the sensitivity of the pathogen under study and the use of stevia leaf extract instead of the purified stevioside.

A thorough bibliographical survey yielded only one study (by our group) on the effects of milk thistle seed extracts on *Fusarium* spp. In that study, composites based on stevioside:silymarin inclusion compounds (in a 5:1 molar ratio) combined with chitosan oligomers in hydroalcoholic solution or in ChCl:urea deep eutectic solvent media were assayed. EC_90_ values of 1241 and 830 µg·mL^−1^ (without and with silver nanoparticles, respectively) were obtained in the first dispersion medium, and 394 and 327 µg·mL^−1^ (without and with silver nanoparticles, respectively) in the second dispersion medium [20]. The values reported herein for the stevioside–milk thistle seed extract conjugate complex (1:1 molar ratio) are noticeably better and involve a much simpler preparation procedure.

Concerning the efficacy of gallic acid (taken as a reference in this study) against *Fusarium* spp., Nguyen et al. [30] tested the antifungal activity of gallic acid from *Terminalia nigrovenulosa* Pierre bark against *F. solani*, reporting an 81% rate of disease suppression for a dose of 1000 μg·mL^−1^. Although, in this case, differences in the efficacy of gallic acid may be ascribed to different *Fusarium* species, conflicting results are observed upon comparison with the work by Pani et al. [17]. In this latter study, the EC_50_ values for the different polyphenols assayed against *F. culmorum* were not reported, but they found that fungal growth was only slightly inhibited when 1.5 mM gallic acid (255 mg·mL^−1^) was added to the liquid culture, while at 1000× lower concentrations, 80% inhibition was observed in our study. Even if the prediction of the antifungal susceptibility of a single strain is difficult (see [31] and references therein), provided that the *Fusarium* spp. do not have normal minimum inhibitory concentration (MIC) and minimum effective concentration (MEC) distributions, the difference in intrinsic resistance between the two strains is striking. 

It is worth noting that the synergy factors for the stevioside–milk thistle seed extract and the stevioside–gallic acid conjugate complexes were almost identical (1.43 and 1.41, respectively), which suggests that the GAE in milk thistle seed extracts was estimated in an accurate manner and that its poorer solubility problem (as compared to that of gallic acid), which could be responsible for its lower efficacy when used alone, would be solved by conjugation with the glycoside.

Regarding the grain protection trials, it is worth noting that the application of wheat straw vinegar (the main components of which are phenolics and acetic acid), diluted 200-fold, has been reported to significantly decrease wheat *F. graminearum* infection rates and DON content by 66% and 69%, respectively [32]. In a similar fashion, Scaglioni et al. [33] found that microalgal phenolic extracts of *Nannochloropsis* sp. and *Spirulina* sp. were able to control *F. graminearum* development and limit DON contamination (−97% for *Nannochloropsis* and −62% for *Spirulina*). Hence, the performance of the stevioside–milk thistle seed extract conjugate complex was better and comparable to those attained with several essential oils (from oregano, cinnamon, palmarosa, orange, spearmint, verbena, fennel, and rosewood) by Perczak et al. [34].

As regards the seedling tests, the absence of significant differences in the germination rates between the negative control and the noninoculated seeds treated with the conjugate complex suggests that a phytotoxic effect of the formulation could be discarded at the concentration used in this experiment. The disease severity results were consistent with the findings of Wiwart et al. [35], who reported that the response to *F. culmorum* infection was weaker in winter wheat than in *T. polonicum* breeding lines and Kamut. In relation to the absence of statistically significant differences in enzymatic activity, in the case of PAL, it may be readily explained because it would only be evident 2 days after inoculation [36]. In the case of PPO, POD, and APX, our negative results would be consistent with those reported by Orzali et al. [36], who found that the enzymes did not vary significantly among treatments in the case of durum wheat treated with chitosan against *F. graminearum*.

### 3.2. Mechanism of Action

The antifungal action of the composites may be ascribed to both the phenolic compounds in milk thistle seed extracts and to stevioside. With regard to the former, the inhibitory behavior arises from their ability to disrupt the integrity of the plasma membrane and mitochondrial dysfunction, inducing metabolic stagnation [37]. Gallic acid exhibits both antioxidant as well as pro-oxidant characteristics, which turns it into an efficient apoptosis-inducing agent [38]. In turn, milk thistle seed extracts target the plasma membrane. As explained by Yun et al. [39,40], silymarin increases the permeability of and physically perturbs the plasma membrane, resulting in its malfunction (with depolarization, K^+^ leakage, and decrease in membrane fluidity), and induces intracellular reactive oxygen species (contributing to the peroxidation of membrane lipids). Moreover, it is known that phenolic acids and flavonoids bind to adhesins [41], i.e., proteins located on the surface of fungal cells that allow fungi to colonize various substrates and to bind to host tissues.

Concerning the antifungal activity of stevioside (the major constituent of *S. rebaudiana* extract), it is mediated by the isosteviol beta OH derivatives (7*β*-, 11*β*-, 12*β*-, and 17*β*-hydroxyisosteviols) [42] that result from the stevioside–fungi interaction. In a first step, fungi-mediated hydrolysis of stevioside leads to the aglycon steviol or its rearranged derivative, isosteviol (*ent*-16-ketobeyeran-19-oic acid). Subsequently, fungi metabolize isosteviol into beta OH derivatives through a stereoselective introduction of OH groups at positions C-7, C-11, C-12, and C-17, as well as C-1, C-6, C-15, and further ketonization at the C-1 and C-7 positions [43]. The evidence suggests that the action mechanism of these molecules is related to the uncoupling of mitochondrial oxidative phosphorylation or the permeabilization of the cell membrane as it occurs with 7*β*-hydroxy-kaurenoic acid (kaurens and beyerans are closely related) [44].

**Figure 6:**
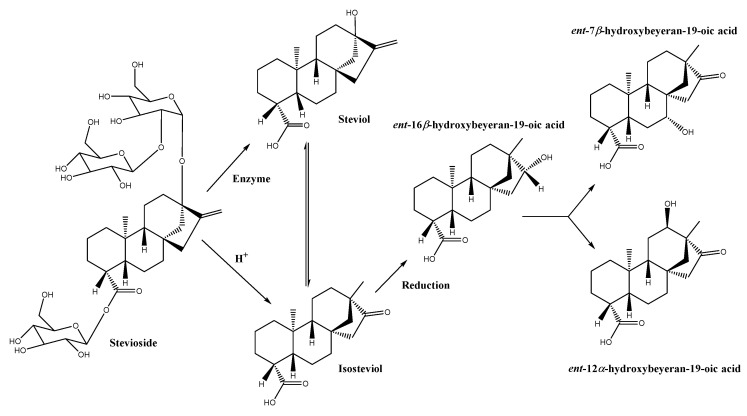
Structures of diterpenoids isolated from microbial transformations of isosteviol. Adapted with permission from Lin et al. [47].

In the case of *F. verticilioides* (the only species of the *Fusarium* genus studied in the literature), the biotransformation of isosteviol leads to *ent*-7*β*-hydroxy-16-ketobeyeran-19-oic and *ent*-12*α*-hydroxy-16-ketobeyeran-19-oic as main metabolites (Figure 6) [45,46]. The selective hydroxylation of the ketobeyeran nucleus of these metabolites is similar to the one which has led to the enhanced activity of the metabolites identified by Lin et al. [47], with hydroxylation at the 7*β*-, 12*α*-, and 14*α*-positions and oxidation of the skeleton at the 16-position. There is a presumption that some of these metabolites may be involved in the transformation of isosteviol by *F. culmorum* and that the observed antifungal efficacy may be referred to them.

## 4. Materials and Methods

### 4.1. Reagents and Fungal Isolate

Stevioside (CAS 57817-89-7, 99%) was purchased from Wako Chemicals GmbH (Neuss, Germany). Powdered milk thistle extract (CAS 22888-70-6; 30% silybin, 98% HPLC) was purchased from KingHerbs Ltd. (Yongzhou, Hunan, China). Gallic acid (CAS 149-91-7; anhydrous, for synthesis), ethanol (CAS 64-17-5; ACS reagent grade), and Tween^®^ 20 (CAS 9005-64-5) were supplied by Sigma-Aldrich/Merck KGaA (Darmstadt, Germany). Potato dextrose agar (PDA) and potato dextrose broth (PDB) were supplied by Becton, Dickinson & Company (Franklin Lakes, NJ, USA).

*Fusarium culmorum* strain CECT 20486 was supplied by the Spanish Type Culture Collection (CECT; Valencia, Spain).

### 4.2. Preparation of the Bioactive Solutions

One treatment based only on stevioside, two treatments based only on polyphenols (either milk thistle extract or pure gallic acid), and their combinations were assayed. It should be clarified that gallic acid was tested as a reference, as materials of plant origin are usually characterized by high variability of phytochemical composition as a result of both genetic variability and environmental variability (influence of weather and soil fertility on the content of active substances).

Ultrasonication-assisted aqueous biphasic system separation was used to prepare the stevioside–polyphenol conjugate complexes in a 1:1 molar ratio. Briefly, 50 mL of an aqueous solution of stevioside (126 mg, MW = 804.87 g·mol^−1^, 0.156 mM) was mixed with a 50-mL ethanol solution of either gallic acid (26.5 mg, MW = 170.12 g·mol^−1^, 0.156 mM) or milk thistle seed extract (250 mg, out of which 75 mg of silybin, MW = 482.44 g·mol^−1^, 0.156 mM). The polyphenol contents in the initial solutions were 0.26 and 0.29 mgGAE·gDW^−1^, respectively, as confirmed by Singleton’s method, using the Folin–Ciocalteu reagent, in good agreement with Mhamdi et al. [25]. The solutions were sonicated with a probe-type UIP1000hdT ultrasonicator (Hielscher, Teltow, Germany; 1000 W, 20 kHz) for 15 min, keeping the temperature below 60 °C.

### 4.3. In Vitro Tests of Mycelial Growth Inhibition

The biological activity of the treatments was determined using the agar dilution method, incorporating aliquots of stock solutions into the PDA medium to provide final concentrations of 32, 64, 96, 128, 192, 256, 384, and 512 µg·mL^−1^. These concentrations, instead of the usual ones defined in CLSI or EUCAST standard antifungal susceptibility testing procedures, were chosen so as to have a true quantitative effect, provided that no growth was observed at concentrations ≥500 µg·mL^−1^. Mycelial disks of the pathogen (5 mm in diameter) from the edges of a 7-day old culture were transferred to plates filled with these media (three plates per treatment and concentration, with two repeats), using plates containing only the PDA medium as the control. 

Radial mycelial growth was determined by the calculation of the average of two perpendicular colony diameters for each replicate. Mycelial growth inhibition for each treatment and concentration after 7 days of incubation, at 25 °C in the dark, was calculated according to the following formula: $\left(\left(d_{c}-d_{t}\right)/d_{c}\right)\times 100$, where *d_c_* is the average diameter of the fungal colony in the control, and *d*_t_ is the average diameter of the fungal colony treated with the tested composite. 

Results were also expressed as EC_50_ and EC_90_ effective concentrations, estimated by fitting the radial growth inhibition values (%) with a DoseResp function, using an orthogonal distance regression (ODR) algorithm.

### 4.4. Preparation of Inoculum and Inhibition of Colonies Formation

*F. culmorum* conidial suspensions were obtained according to the procedure described by Khan et al. [48], with minor modifications. Conidia were harvested from 7-day-old PDB cultures (200 mL cultures, incubated at 25 °C under constant stirring at 140 rpm in a Labolan (Esparza de Galar, Navarra, Spain) orbital stirrer incubator (model ECOLAN 60). The conidial suspension was filtered through two layers of sterile muslin to remove hyphal fragments, and the conidial concentration was first determined using a Weber Scientific International Ltd. (Teddington, Middlesex, UK) Neubauer chamber and then adjusted to a final concentration of 5 × 10^4^ or 1 × 10^5^ conidia·mL^−1^ (depending on the experiment to be conducted afterwards), adding 0.2% Tween^®^ 20.

In vitro tests aimed at determining the inhibition rate percentage of the number of colonies formed were carried out according to the procedure reported by Kheiri et al. [49]. Briefly, 0.5 mL of the conidial suspension (5 × 10^4^ conidia·mL^−1^) was mixed with different concentrations (128, 256, and 512 µg·mL^−1^) of the solution of stevioside, stevioside–milk thistle seed extract, or stevioside–gallic acid conjugate complexes to a final volume of 2 mL. The conidial suspension was also prepared with distilled water and 0.5% *v*/*v* acetic acid aqueous solution as the control. The resulting solutions were incubated at 25 °C for 24 h. Aliquots of 50 µL of each dilution were spread on PDA with a Drigalski spatula and incubated at 25 °C, counting the number of colonies formed after 5 days. The test was repeated twice, and each treatment had 3 replicates. The percent inhibition rate was estimated as

(1) $$\%\;\mathrm{Inhibition}\;\mathrm{rate}=\frac{\mathrm{number}\;\mathrm{of}\;\mathrm{colonies}\;\mathrm{formed}\;\mathrm{in}\;\mathrm{control}\;\mathrm{plate}\;–\;\mathrm{number}\;\mathrm{of}\;\mathrm{colonies}\;\mathrm{formed}\;\mathrm{in}\;\mathrm{treated}\;\mathrm{plates}}{\mathrm{number}\;\mathrm{of}\;\mathrm{colonies}\;\mathrm{formed}\;\mathrm{in}\;\mathrm{control}\;\mathrm{plate}}\times 100$$

### 4.5. Effect of Conjugate Complex on DON Production and DON Determination

The effect of the stevioside–milk thistle seed extract conjugate complex on the growth of *Fusarium* fungi on Kamut (*Triticum turgidum* subsp. *turanicum* (Jakubz.) Á.Löve) and winter wheat (*Triticum aestivum* L.; cv. Sofru) grain was investigated using the method described by Perczak et al. [34]. Briefly, 5 mL of the conjugate complex solution, at a concentration of 512 µg·mL^−1^, was mixed with 25 g of sterile grain in an Erlenmeyer flask. The mixture was vigorously stirred, and three rings (6 mm) of solid culture of *F. culmorum* were then added to each Erlenmeyer flask and further mixed. Solutions of Tween^®^ 20 and deionized water were added to the negative and positive control samples (without the addition of the bioactive compound). Next, the prepared mixtures were stored in the dark at 25 °C for 28 days. After incubation, samples were dried, milled, homogenized, and prepared for chromatographic analysis. 

The organic extracts were obtained by soaking of the samples in a mixture of water, methanol, and acetonitrile in a 10:10:30 *v*/*v* ratio, followed by sonication for 15 min in five 3-min periods. The supernatant solution was filtered with Whatman nº 4 paper and stored at 4 °C until the analytical determinations were carried out. The determination of DON was outsourced to the Laboratorio de Técnicas Instrumentales (University of Valladolid, Valladolid, Spain). The analyses were carried out according to the procedure recommended by Jerome Jeyakumar et al. [50], using a QTOF X500R (Ab Sciex Spain S.L., Madrid, Spain) mass spectrometer coupled to a 2D-UHPLC EXION LC series system and taking a deoxynivalenol solution (CAS 51481-10-8; certified reference material; Sigma-Aldrich, Madrid, Spain) as a reference.

### 4.6. Seedling Tests

The potential ability of the conjugate complex treatment to induce resistance in seedlings of Kamut and winter wheat against *F. culmorum* was evaluated following the methodology reported by Orzali et al. [36], with minor modifications. Kamut and winter wheat grains were first surface-sterilized for 3 min by immersion in 2% NaOCl and then rinsed with water. The seed treatments (100 seeds per treatment) were performed by immersion in 100 mL of conjugate complex solution (at a concentration of 512 µg·mL^−1^, with 0.2% Tween^®^ 20) at room temperature, under vigorous stirring for 1 h. Distilled water with 0.2% Tween^®^ 20 was used in the negative and positive controls. The seeds were then air-dried for 30 min, and inoculated by immersion in 100 mL of the 1 × 10^6^ conidia·mL^−1^ suspension, with 0.2% Tween^®^ 20, for 30 min. The seeds were finally air-dried again for 30 min. 

The physiological quality of the seeds for each treatment (negative control, positive control, phytotoxicity test, and treatment with the bioactive formulation) was evaluated by germination, as described in the International Rules for Seed Testing. For each treatment, 3 replicates of 100 seeds were placed on glass plates, using the between-paper method, and kept under constant humid conditions. Germination was evaluated after 4 days in such a way that a seed was considered germinated if it produced a well-developed seedling, with three roots and a shoot present. 

The efficacy of the antifungal treatment was then assessed by planting the seeds in pots filled with autoclaved peat-based substrate, with a procedure similar to those described by Lozano-Ramírez et al. [51] and Koch et al. [52]. Seedlings were grown in greenhouse conditions at 25 °C, extracted after 2 weeks, and symptoms in the roots and the internode were visually evaluated using a 0 to 5 scale [53]. The protection function was described using the disease index [54] (Equation (2)) and by applying Abbott’s formula to determine the efficacy percentage [55] (Equation (3)):

(2) $$\mathrm{Disease}\;\mathrm{index}\;\left(\mathrm{DI}\right)=\frac{\sum^{\;}\left(\mathrm{disease}\;\mathrm{grade}\;\times \;\mathrm{no}.\;\mathrm{of}\;\mathrm{plants}\;\mathrm{in}\;\mathrm{each}\;\mathrm{grade}\right)}{\left(\mathrm{total}\;\mathrm{no}.\;\mathrm{of}\;\mathrm{plants}\right)\;\left(\mathrm{highest}\;\mathrm{disease}\;\mathrm{grade}\right)}\times 100$$

(3) $$\mathrm{Efficacy}\;\mathrm{percentage}=\left(\frac{\%\;\mathrm{infection}\;\mathrm{control}-\%\;\mathrm{treated}\;\mathrm{infection}}{\%\;\mathrm{infection}\;\mathrm{control}}\right)\times 100$$

The enzymatic activities of some enzymes involved in defense mechanisms (either used as stress markers, i.e., guaiacol peroxidase, ascorbate peroxidase, or involved in phenylpropanoid metabolism, i.e., polyphenol oxidase and phenylalanine ammonia lyase), were extracted from the hypocotyls of the wheat seedlings and analyzed according to the procedures described by Orzali et al. [36].

### 4.7. Statistical Analyses

Data were subjected to analysis of variance (ANOVA) in IBM SPSS Statistics v.25 software (IBM, Armonk, NY, USA) after checking normality and homoscedasticity assumptions. The Tukey’s HSD test at a 0.05 probability level (*p* < 0.05) was used for the post hoc comparison of means.

## 5. Conclusions

The potential synergistic effect in terms of antifungal activity resulting from the combination of aqueous solutions of stevioside and alcoholic solutions of milk thistle seeds extracts (rich in polyphenols), preconized as the working hypothesis, was evidenced against *F. culmorum* (with a synergy factor of 1.4). Silybin enhanced the antifungal efficacy of stevioside, which, on the other hand, proved to be sufficiently bioactive: EC_50_ and EC_90_ values of 123.2 and 159.7 µg·mL^−1^, respectively, were obtained for the stevioside–milk thistle seed extract conjugate complex vs. 155.9 and 221 µg·mL^−1^, respectively, for stevioside. The choice of the 1:1 molar ratio noticeably improved the antifungal activity as compared with previously reported inclusion compounds of stevioside and polyphenols in a 5:1 molar ratio. Full inhibition of colony formation was confirmed at 256 µg·mL^−1^ for the conjugate complex-based treatment, which was also tested for grain protection at storage, finding that at a concentration of 512 µg·mL^−1^, it fully inhibited fungal growth and thus prevented trichothecene production. Assays conducted with Kamut and winter wheat seed tests indicated that the bioactive formulation at 512 µg·mL^−1^ reduced seedling root rot (by 71% and 60%, respectively) and did not affect the germination rate. These results suggest that these stevioside–*S. marianum* extract conjugate complexes may hold promise as antifungal agents for FHB treatment. 

## 6. Patents

The work reported in this manuscript is related to Spanish patent P201731489.

## Figures and Tables

**Table 1:** Effective concentrations that inhibited mycelial growth by 50% and 90% (EC_50_ and EC_90_, respectively).

Concentration (µg·mL^−1^)	Stevioside	Milk Thistle	Gallic Acid	Stevioside + Milk Thistle	Stevioside + Gallic Acid
EC_50_	155.92 ± 26.25	170.08 ± 22.82	150.16 ± 20.13	123.15 ± 14.79	125.33 ± 17.22
EC_90_	221.01 ± 52.73	237.01 ± 42.74	228.94 ± 42.58	159.70 ± 24.64	160.03 ± 29.39
